# Current Efforts in the Development of Vaccines for the Prevention of Zika and Chikungunya Virus Infections

**DOI:** 10.3389/fimmu.2020.00592

**Published:** 2020-04-09

**Authors:** Sabrina Schrauf, Roland Tschismarov, Erich Tauber, Katrin Ramsauer

**Affiliations:** Themis Bioscience GmbH, Vienna, Austria

**Keywords:** Zika virus, Chikungunya virus, vaccine development, arbovirus, emerging diseases

## Abstract

Arboviruses represent major challenges to public health, particularly in tropical, and subtropical regions, and a substantial risk to other parts of the world as respective vectors extend their habitats. In recent years, two viruses transmitted by Aedes mosquitoes, Chikungunya and Zika virus, have gathered increased interest. After decades of regionally constrained outbreaks, both viruses have recently caused explosive outbreaks on an unprecedented scale, causing immense suffering and massive economic burdens in affected regions. Chikungunya virus causes an acute febrile illness that often transitions into a chronic manifestation characterized by debilitating arthralgia and/or arthritis in a substantial subset of infected individuals. Zika infection frequently presents as a mild influenza-like illness, often subclinical, but can cause severe complications such as congenital malformations in pregnancy and neurological disorders, including Guillain–Barré syndrome. With no specific treatments or vaccines available, vector control remains the most effective measure to manage spread of these diseases. Given that both viruses cause antibody responses that confer long-term, possibly lifelong protection and that such responses are cross-protective against the various circulating genetic lineages, the development of Zika and Chikungunya vaccines represents a promising route for disease control. In this review we provide a brief overview on Zika and Chikungunya viruses, the etiology and epidemiology of the illnesses they cause and the host immune response against them, before summarizing past and current efforts to develop vaccines to alleviate the burden caused by these emerging diseases. The development of the urgently needed vaccines is hampered by several factors including the unpredictable epidemiology, feasibility of rapid clinical trial implementation during outbreaks and regulatory pathways. We will give an overview of the current developments.

## Introduction

Less than 20 years ago Chikungunya and Zika virus were endemic on the African continent and only caused sporadic and small, local outbreaks (1, 2). Several factors contributed to a global spread of these infections, including deforestation bringing humans close to the zoonotic reservoir of potential human pathogens, climate change leading to expansion of the animal vector habitats, economic expansion, and globalization in general (3–5). Chikungunya and Zika virus belong to a group of arthropod-borne viruses (Arboviruses) that are transmitted by the *Aedes* species mosquitos, in most cases by *Aedes aegypti* and *Aedes albopictus*. Arboviruses are a major threat to human health. In addition to CHIKV and ZIKV, the family of these viruses comprise different human pathogens that can cause acute infections including Dengue Virus (DENV), Yellow Fever Virus (YFV), West Nile Virus (WNV), Japanese Encephalitis (JEV), Ross River Virus (RRV), and Eastern Equine Encephalitis virus (EEEV). Over 100 arboviruses are known to date that can cause infections in humans, and it is a widely accepted belief that many more such viruses remain to be discovered (3). Transmission to humans is mediated by the bite of an infected mosquito and the infection can cause a range of clinical outcomes, from asymptomatic to encephalitis (WNV, JFV, and ZIKV) or fatal hemorrhagic fever (YFV and DENV).

Zika Virus (ZIKV) is a flavivirus of African origin which was first identified in a rhesus monkey in the Zika Forest in Uganda 1947 (6). The virus is primarily transmitted by mosquitos from the genus *Aedes* (7, 8). In addition to the vector borne transmission, sexual transmission as well as transmission via blood transfusion is a likely route of infection. An infamous feature of ZIKV infections is the vertical transmission from mother to child during pregnancy (9, 10) that can lead to abnormal brain development of the fetus (11, 12). Such fetal phenotypes have been described as congenital ZIKV syndrome (13). In nature, the virus is maintained primarily in a sylvatic cycle between non-human primates (NHP) and mosquitoes (14).

Chikungunya virus (CHIKV) is an alphavirus transmitted by mosquitoes that causes a febrile disease referred to as Chikungunya fever. Like ZIKV, CHIKV was first isolated in Africa, in Tanzania in 1952. The disease is characterized by high, transient fever, polyarthralgia, and skin manifestations (15). While most patients recover from acute Chikungunya fever a substantial subset of people experience a transition to severe chronic arthralgia and arthritis that can last for months or years (16, 17). Besides moving between humans and mosquitoes, the virus can also exist in purely enzootic cycles between non-human primates and mosquitoes (18).

ZIKV and CHIKV have gathered increased interest in recent years due to several massive outbreaks. Climate change and increased travel activities have led to unprecedented spread of these viruses, particularly throughout tropical and subtropical regions, but also to temperate zones. Autochthonous transmission of CHIKV was reported in several European countries including Spain, France and Italy (19, 20). In November 2019 the first locally acquired cases of Zika were reported in Europe (21, 22). In addition to the transmission of ZIKV by an animal vector the disease can also be transmitted sexually, which increases the risk of disease in emergence in previously non-endemic areas (23). Generally, the virus was introduced by travelers returning from affected areas, stressing the importance for the development of effective vaccines.

Vaccination is the most effective defense against unpredictable outbreaks of emerging infectious diseases. Currently, there is no treatment or vaccine available to prevent CHIKV or ZIKV disease. Here, we give a brief overview on the molecular virology, epidemiology, pathogenesis and the immune response to ZIKV and CHIKV, followed by a summary of past and current efforts to develop vaccines against these diseases. Finally, we will discuss the current regulatory and policy framework that will facilitate and accelerate the development of a ZIKV and a CHIKV vaccine.

### Molecular Virology and Epidemiology

#### Zika Virus

**Figure 1:**
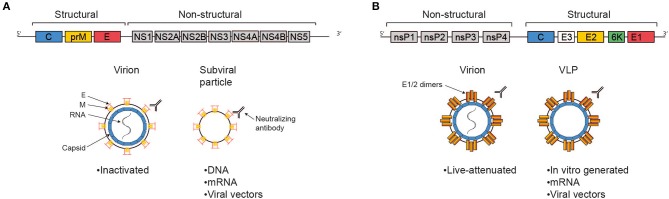
Antigens delivered by the most advanced vaccine candidates against ZIKV and CHIKV. Schematic representations of the viral genomes are shown at the top, simplified virions, VLPs, and subviral particles are shown below, as indicated. The text boxes indicate which of the more advanced vaccine candidates deliver antigen in these fashions. **(A)** The Zika genome encodes the structural genes C, prM, and E and the non-structural genes NS1-5. The E protein represents the immunodominant antigen, neutralizing antibodies against which are protective. Advanced vaccine candidates differ in their platform technology, but ultimately all present immunodominant antigen either in the context of a full virion or a sub-viral particle. **(B)** The Chikungunya genome consists of the non-structural genes nsP1-4 and a subgenomic ORF encoding the structural genes C, E3, E2, 6K, and E1. Protection is mediated via the induction of neutralizing antibodies, epitopes for which are predominantly found in E2 and, to a lesser extent, E1. The most advanced candidate vaccines are based on different platform technologies, but ultimately all present E1/2 either in the context of a full virion or on VLPs.

The ZIKV genome consists of single stranded positive sense RNA of about 11 kB in length which harbors one single open reading frame flanked by 5′ and 3′ non-coding regions (Figure 1A). Translation yields one single polyprotein that is co- and post-translationally processed into three structural proteins—capsid (C), precursor of membrane (prM) and envelope (E)—and seven non-structural proteins (NS1, NS2A, NS2B, NS3, NS4A, NS4B, NS5) by viral and host cell proteases. The ZIKV virion is a spherical, enveloped virus particle with a diameter of approximately 50 nm (24, 25). The nucleocapsid generated through interaction of the RNA genome with multiple copies of the capsid protein is engulfed by a host cell derived lipid bilayer in which the two other structural proteins prM and E are embedded via transmembrane helices. The non-structural proteins are essential for RNA replication and assembly (26). During the maturation process of the virions, protein M is generated from the precursor protein by proteolytic furin cleavage (27). Infectious mature virions carry the E proteins as homodimers aligned in parallel to the virion surface (28). The E protein mediates cellular attachment, entry and fusion of the viral and the host cell membrane (29). Moreover, this protein represents the immunodominant antigen of all flaviviruses. Neutralizing antibodies triggered by flaviviral infections are raised against this specific membrane protein (30).

ZIKV exhibits a very broad tissue tropism in humans ranging from the brain, skin and immune cells to placenta, testis, kidney, and retina. The virus enters the cell via clathrin-dependent endocytosis following interaction of the receptor protein E with cell surface receptors. Multiple cell surface receptors facilitate viral entry, among these are tyrosine-protein kinase receptors AXL, Tyro3, DC-SIGN, and TIM-1 (31–33). Upon entry by endocytosis, low pH within the vesicles triggers conformational changes of protein E dimers to trimers and subsequently the exposition of the fusion peptide, further resulting in fusion of the viral membrane with the endosomal target membrane (34). The nucleocapsid disassembles and releases the viral genome that subsequently undergoes replication, and translation in intracellular membrane-associated compartments on the surface of the endoplasmatic reticulum (ER) (35). During translation, the membrane proteins E and prM are translocated into the lumen of the ER where they interact with each other and form heterodimers. The association of prM/E heterodimers into higher-ordered structures is the driving force for virion budding. Finally, the immature particles are transported through the trans-Golgi network where mature infectious virus particles are generated upon cleavage of prM into M, subsequent resolution of the heterodimers and formation of fusion-competent E protein dimers (36).

Following the discovery of ZIKV in 1947, only sporadic cases of natural ZIKV infection of human beings were reported over a period of 60 years. In 2007, the first large ZIKV outbreak occurred on Yap Island (Micronesia) during which three quarters of the population were affected within a few months (37). ZIKV infection was described as a mild self-limiting disease with symptoms such as rash, fever, conjunctivitis, arthralgia and arthritis (38). The next ZIKV epidemic outburst occurred in French Polynesia in 2013 which was characterized by a high attack rate and associated for the first time with the development of Guillain-Barré syndrome (39, 40). Two years later the virus hit South America, with Brazil particularly affected (41, 42). During this outbreak, a dramatic increase in cases of Guillain-Barré Syndrome (43) and congenital neurological disorders such as microcephaly in infants born from women infected with the virus during pregnancy were recorded (44–46). Up to now, autochthonous transmission of ZIKV was documented in over 80 countries world-wide. Recently, locally acquired ZIKV infection has been documented for the first time in France, Europe (21, 47).

Zika virus can be grouped into two major lineages, African and Asian. Strains identified during the epidemic in South America all belong to the Asian lineage and have high degrees of similarity with strains from Polynesia (48). Distinct changes in the genetic sequence may have contributed to the dramatic increase in pathogenicity of ZIKV since 2007 (49, 50). Among mosquito-borne flaviviruses, ZIKV is most closely related to the four serotypes of DENV which may impact pathogenesis due to cross-reactive antibodies (51).

#### Chikungunya Virus

CHIKV is a single-strand, positive-sense RNA alphavirus belonging to the *Togaviridae* family. The genome is about 12 kb, encoding four non-structural genes (nsP1-4) and five structural genes (C-E3-E2-6K-E1) expressed from a subgenomic RNA (Figure 1B). Virions consist of the genome packed into the nucleocapsid and are enveloped with a host-cell derived lipid bilayer (52). The surface of the mature virion is covered in trimeric spikes consisting of E1/E2 dimers, which are essential for receptor binding and membrane fusion (53). CHIKV can infect a wide variety of cells, including keratinocytes, fibroblasts, osteoblasts, chondrocytes, skeletal muscle cells, and monocytes/macrophages (54). Virions are taken up via clathrin-mediated endocytosis (and potentially other pathways, depending on cell type). The engaged receptors are incompletely understood, besides convincing data on the importance of MXRA8, several other candidates have been suggested, including prohibitins and glycosaminoglycans (55, 56). Upon uptake, E1 and E2 undergo conformational changes caused by endosomal pH, resulting in membrane fusion and release of the nucleocapsid into the cytoplasm (55). The non-structural proteins get translated and processed to form an initial viral replication complex, producing a negative-sense RNA intermediate serving as the template for further synthesis of the 49S full genomic RNA and the 26S subgenomic RNA (encoding the structural proteins). Structural proteins are then translated and post-translationally modified, capsid and the 49S RNA are assembled to form the nucleocapsid in the cytoplasm, and mature virions are assembled at the cell membrane followed by budding (57).

CHIKV was first described after a 1952/1953 outbreak in Tanzania. Several outbreaks of varying size were reported in Africa and Asia in the following decades, with the first report in Asia occurring in the Philippines in 1954 (15). After a particularly large outbreak in Kenya in 2004, the virus spread to previously naïve populations on islands in the Indian Ocean, including Comoro, La Reunion and the Seychelles, and further to India, ushering in a series of unprecedentedly large chikungunya outbreaks (58, 59). Travelers introduced the virus to previously non-endemic regions harboring vector populations. In Europe the first autochthonous outbreak was described in 2007 in Italy. An explosive outbreak in the Americas started at the end of 2013 on the island of St. Martin. Chikungunya spread rapidly through the Americas, affecting 26 islands and 14 mainland countries and causing over a million reported cases within a year. By 2015, 1.7 million cases had been reported from 45 countries or territories reporting to the Pan American Health Organization (60). Recently, the number of reported CHIKV cases in the Americas went down significantly. However, close to 100.000 cases were reported from Brazil in 2019 (61). Other countries with transmissions reported in the Americas were Bolivia, Nicaragua, and Venezuela. In addition, outbreaks have been reported in Asia from Thailand, Malaysia, and India in 2019. Also, several African countries were affected including Ethiopia, Republic of Congo, and Sudan (November 22th 2019, ECDC https://www.ecdc.europa.eu/en/chikungunya-monthly).

Chikungunya viruses have been divided into three genotypes based on phylogeny: West African (WAf), East Central South African (ECSA), and Asian genotype, with the Asian genotype likely derived from ECSA (60). During the outbreak on La Reunion in 2006, phylogenetic analysis revealed that the causative virus (of ECSA descent) had acquired a new mutation in the gene coding for E1 that favored infectivity in *Aedes albopictus* (62). This genotype is now referred to as the Indian Ocean Lineage (IOL). Several studies have found that antibodies raised by one genotype are cross-reactive against all others, leading to the widely held opinion that CHIKV comprises a single serotype (63–65).

### Pathogenesis and Clinical Manifestations

#### Zika Virus

Originally, ZIKV infection in humans was described as mostly asymptomatic or mild with self-limiting flu-like symptoms in about 20–25% of infected people following an incubation period of 4–10 days (37, 66). These non-specific symptoms may include transient low-grade fever, itchy maculopapular rash, arthritis or arthralgia, and non-purulent conjunctivitis; to a lesser extent, retroorbital-pain headache, myalgia, edema and vomiting. Most of the symptoms resolve within a week, except arthralgia which may persist up to 4 weeks (67).

The recent, large outbreak in the Americas drastically showed the ZIKV infection is also associated with severe disease. The virus can cause thrombocytopenia, subcutaneous bleeding (68) and multi-organ failure (69). Besides that, infection of the eye in adults can lead to the development of uveitis (70). Rarely, the virus induces encephalitis and meningitis in this age group (71). Importantly, due to the high number of cases, also rare features of ZIKV infection were observed. During pregnancy, ZIKV infection affects the fetus and causes malformations of the brain such as microcephaly and other neurological disorders. Congenital ZIKV infection may also lead to hearing loss, ocular anomalies well as intrauterine growth restrictions and/or fetal demise. These alarming features have characterized the recent outbreaks in America. Pre-and post-natal cases have highlighted the direct link between ZIKV infection during pregnancy and the increased risk of microcephaly and congenital abnormalities (1). One prospective study in Brazil which analyzed symptomatic ZIKV-infected pregnant women revealed that almost 30% of all fetuses showed abnormal intrauterine development (44). The difference between disease outcome in adults and infants may be explained by the fact that the virus preferentially infects neural progenitor cells (NPCs) leading to dysregulation of their cell cycle and subsequent apoptosis (72). Recently, Ferraris et al. provided some mechanistic insight by showing that ZIKV infection of NPCs induces early activation of the Notch pathway leading to impaired neurogenesis (73).

ZIKV infection in adults has been associated with the development of Guillain-Barré syndrome (GBS), an autoimmune disease causing peripheral nerve damage by the immune system that leads to muscle weakness and paralysis of the limbs (74, 75). The incidence of GBS during the French Polynesian Outbreak was about 20-fold higher than baseline levels. A case-control study performed in French-Polynesia supported the direct connection between ZIKV infection and GBS development as almost all patients (41 of 42) with GBS had detectable anti-ZIKV IgM/IgG levels and all showed neutralizing antibodies against ZIKV (40). Interestingly, ZIKV-induced GBS seems to be transient and most patients have recovered fully.

#### Chikungunya Virus

Chikungunya fever symptoms commence after an incubation period of 2–12 days following transmission from an infected mosquito. The majority of patients then enter the acute phase of Chikungunya fever, characterized by high fever, severe myalgia and arthralgia, and rashes as the most common manifestations. The rate of asymptomatic infection seems to vary from 4 to 28%, which depends on the CHIKV lineage and age of the patient, with higher rates of asymptomatic disease in children (76). The infection is usually non-fatal and self-limiting, and the symptoms resolve within a few days, but a substantial subset of patients experience transition of the disease into a chronic phase, described in more detail below (15). Joint pain is most frequently bilateral, symmetric, and primarily observed in the extremities (58). Synovitis and joint swelling are frequently reported with large joint effusions in 15% of individuals infected with Chikungunya (77, 78). Macropapular and macular rashes are observed in 10–40% of patients, are limited to the trunk and extremities in most patients and are mostly transient. A wide variety of less common symptoms, including digestive abnormalities, lymphadenopathy, and ocular complication have been described (54), these cases are summarily described as atypical acute Chikungunya disease (79). Some patients, usually elderly, infants or individuals with pre-existing comorbidities, experience severe acute Chikungunya fever. Frequently observed complications include encephalitis, hepatitis, myocarditis, renal failure as well as respiratory disorders. In these cases, CHIKV infection can be lethal, albeit with a relatively low frequency [e.g., under 1 in 1,000 patients during the La Reunion outbreak (80)]. Of note, some reports suggest that these numbers may have been previously underreported or might be elevated in recent outbreaks (81, 82). The percentage of patients developing chronic Chikungunya fever varies between outbreaks, with up to 60%, as described during the La Reunion outbreaks (83). Chronic Chikungunya is characterized by persistent or relapsing severe poly-arthralgia, mainly described in small joints of hand, feet and ankles, which can last for months or even years and severely impacts patient quality of life (78). Besides the effect on mobility and well-being there is a major economic burden to the affected health systems associated with CHIKV outbreaks (2).

### Animal Models and Immune Responses

#### Zika Virus

As ZIKV infections are able to induce versatile symptoms and diseases, researchers have put a lot of effort in the development of different animal models to investigate mechanisms of pathogenesis and host immune responses (Table 1). The explosive outbreak in the Americas required availability of animal models to better understand ZIKV pathogenesis and to develop effective vaccines. Within a short time period different mouse and NHP models were developed [reviewed in (84)]. Mice with defects in the interferon system—single and double knockouts of the type I and type II interferon (IFN) receptors on either the 129/SV genetic background (A129 or AG129, respectively) or single type I knockout on C57BL/6 genetic background (IFNAR1^−/−^) or wild-type C57BL/6 mice treated with an IFNAR1 receptor blocking monoclonal antibody– are frequently used to study Zika virus infections in adults. Dependent on the ZIKV strain and the age of the animals, infected mice demonstrate signs of tremor, ataxia, paralysis, conjunctivitis, damage of the central nervous system as well as inflammation of the male reproductive tract. In addition, neonate C57BL/6 mice are a useful model to study mechanisms of pathogenesis as well as neurodevelopmental processes. Pregnant IFNAR1 knockout mice and pregnant C57BL/6 mice treated with anti-IFNAR1 monoclonal antibody represent a good model to investigate ZIKV infection during pregnancy. In these animals, ZIKV induces pathological changes in the placenta, intrauterine growth restriction of the fetuses and fetal brain development defects. Recently, BALB/c mice treated with an anti-IFNAR1 monoclonal antibody as well as neonate BALB/c mice have also been used to model ZIKV infection in adults as well to study neuropathogenesis, respectively (85, 86). NHP models—rhesus, cynomolgus, as well as pigtail macaques—are used to study ZIKV cell and tissue tropism and the adaptive immune response. Infected monkeys develop potent humoral as well as cellular immune responses against ZIKV which protect against subsequent challenge (87). Thus, NHP also represent a suitable model to evaluate the immunogenicity and efficacy of prophylactic vaccines. As the placental barrier, embryogenesis, and fetal development of monkeys very closely resemble humans, ZIKV infection studies are also performed in pregnant rhesus or pigtail macaques.

**Table 1:** Frequently used animal models for ZIKV infection and disease.

**Model organism**		**Advantages**	**Disadvantages**
**IMMUNOCOMPROMISED MICE**			
IFN signaling deficient-mice	IFNI knock out (IFNAR1^−/−^; A129)	• Small animal model (size, generation time, handling, cost, etc.) • Large body of literature, availability of tools and reagents	• Evolutionary distance • Immunodeficient
	IFNI and II knock out (AG129)	• Replicates high viremia, dissemination to multiple organs, ataxia, tremor and paralysis • Study pathogenesis of eye disease	• Replicate only some aspects of ZIKV infection
	C57BL/6 or BALB/c treated with anti-IFNAR1 mAb	• Study pathogenesis and persistence in male reproductive tract • Lethal and non-lethal models available	• Lethality is age-dependent–100% mortality only in very young mice (3–4 weeks-old)
**IMMUNOCOMPETENT MICE**			
Neonate C57BL/6 or BALB/c		• Small animal model (size, generation time, handling, cost, etc.) • Large body of literature, availability of tools and reagents • Key brain development processes occur post-natally • Replicate pathologies of central nervous system • Sub-lethal—study long-term sequelae on survivors	• Evolutionary distance • Replicate only some aspects of ZIKV infection
**IMMUNOCOMPETENT PRIMATES**			
NHP	Rhesus macaques	• Evolutionary proximity—Similar physiology and immune response • Natural host	• Large animal model (size, generation time, handling, cost, etc.)
	Cynomolgus macaques	• Replicate viremia; spread to different organs and body fluids, changes in blood biochemistry and mostly elevated body temperature • Non-lethal	• Ethical constraints of using primates in research
**INFECTION DURING PREGNANCY**			
Pregnant mice	IFNAR1^−/−^	• Small animal model (size, generation time, handling, cost, etc.) • Availability of tools and reagents • Replicates transplacental viral transmission	• Evolutionary distance • Immunodeficient or not fully immunocompetent (SJL mice)
	C57BL/6 treated with anti-IFNAR1 mAb	• Replicate pathological changes to brains of developing fetuses and intrauterine growth restrictions	
Pregnant NHP	Rhesus macaques	• Evolutionary proximity—similar placental barrier and gestational development • Natural host	• Large animal model (size, generation time, handling, cost, etc.)
	Pigtail macaques	• Replicate persistent viremia and transplacental transmission	• Ethical constraints of using primates in research

The innate immune system plays a key role in controlling ZIKV infections as most infections are asymptomatic or mild. ZIKV infection stimulates the interferon system and thus the production of type I (IFN-α, β), type II (IFN-γ), and type III IFNs (IFN-λ1–4) followed by the induction of IFN stimulated genes such as small membrane-associated interferon-inducible transmembrane proteins (IFITMs) that interfere with specific steps of the viral life cycle (31, 88, 89). IFITM1 and IFITM3 for example have both been described to inhibit ZIKV replication (90). In addition to the innate immune response, ZIKV infection also triggers an adaptive immune response that contributes to protection and possibly also to pathogenesis. Studies in mice have shown that CD4^+^ as well as CD8^+^ T cells are both involved in viral clearance (91–94). Despite this protective role, CD8^+^ T cells may also be involved in brain pathogenesis inducing paralysis in mice (95). B-cell mediated immune responses and the production of neutralizing antibodies play an important role in controlling infections (96). These antibodies bind complex epitopes on the virion surface. Studies in mice and non-human primates (NHP) have shown that antibodies alone are sufficient for protection. A passive transfer of IgG from NHP that previously received a formalin-inactivated ZIKV into naïve recipient mice or NHPs protected both species from viremia following ZIKV challenge (97). In addition, depletion of CD4^+^ and/or CD8^+^ T cells in mice prior challenge had no impact on the protective capacity of a DNA vaccine, again highlighting the protective role of the humoral immune response (98). Moreover, administration of human monoclonal antibodies with neutralizing activity are able to prevent replication, maternal–fetal transmission and disease in mice (99). All these studies together suggest that ZIKV-specific neutralizing antibodies represent an immune correlate of protection. Neutralizing antibody titers of >10, as determined by plaque reduction neutralization test, have been accepted as correlates of protection for other flaviviruses such as Japanese encephalitis and Tick-borne encephalitis virus (100–102). It remains to be elucidated whether a similar titer will confer protection against ZIKV infection in humans and whether a T cell response is necessary to initiate an effective B cell response.

The role of cross-reactive antibodies in disease progression is uncertain. Antibodies that bind but do not neutralize potentially affect the disease outcome of the closely related DENV by a phenomenon known as antibody-dependent enhancement (ADE). Antibodies that are developed during the first DENV infection enhance disease burden in the context of a secondary infection with a heterologous DENV serotype (103). Although cross-reactive ZIKV antibodies can enhance ZIKV infection *in vitro*, no signs of ADE have been observed so far *in vivo* (104). Of note, some findings in mice indicate that pre-existing immunity to Dengue and other flaviviruses might contribute to more severe Zika pathogenesis (105, 106).

#### Chikungunya Virus

Much of our knowledge on the immune response to CHIKV is based on results from animal models (Table 2). Neonatal mice are susceptible to CHIKV challenge and have thus been used to study severe acute disease and lethality (107). Adult mice deficient for components of type I interferon signaling, most commonly IFNAR1^−/−^, are similarly susceptible and are frequently used in lethal challenge models (108, 109). Wild-type animals are significantly less prone to develop disease upon CHIKV challenge, but exhibit swelling, arthritis, and transient viremia upon subcutaneous CHIKV infection in the footpad, which has been frequently used to investigate joint involvement and, to some extent, chronic disease. All of these models are limited in the aspects of disease they mirror, but offer the many advantages associated with mouse models, above all the wide availability of transgenics (110). CHIKV infection in NHPs recapitulates many aspects of human disease and can be considered less artificial in that these animals represent natural amplification hosts for the virus in sylvatic transmission cycles. Moreover, they are genetically and physiologically similar to humans. Initial studies performed in the 1950s and 1960s demonstrated that Rhesus macaques infected with CHIKV develop viremia 2–4 days post-infections (dpi), mount a neutralizing antibody response and are protected from reinfection. More recent work has aimed to better characterize CHIKV pathogenesis in Rhesus and Cynomolgus macaques and both have been frequently used to test vaccines or therapeutic antibodies. Macaques represent an excellent model for CHIKV pathogenesis, but studies are limited by the high cost as well as ethical considerations. In addition, the NHP model fails to replicate joint pathologies observed in human disease unless challenged with very high doses of virus (111, 112).

**Table 2:** Frequently used animal models of Chikungunya disease.

**Model organism**	**Advantages**	**Disadvantages**
Neonate mice	• Small animal model (size, generation time, handling, cost, etc.) • Large body of literature, availability of tools and reagents • Transgenic animals • Replicates high viremia and dissemination to multiple organs	• Evolutionary distance • Immunocompromised • Lethal model with limited time window for analysis
IFNI signaling deficient-mice (e.g., IFNAR1^−/−^, IFNAR1^−/−^, IFNGR1^−/−^)	• Small animal model (size, generation time, handling, cost, etc.) • Large body of literature, availability of tools and reagents • Transgenic animals • Replicates high viremia and dissemination to multiple organs	• Evolutionary distance • Immunocompromised • Lethal model with limited time window for analysis
Footpad swelling in wild-type mice	• Small animal model (size, generation time, handling, cost, etc.) • Large body of literature, availability of tools and reagents • Transgenic animals • Replicates viremia, dissemination to tissues and joints close to injection site and selected organs, arthritis-like disease and viral persistence • Non-lethal	• Evolutionary distance • Fails to replicate dissemination to all organs affected in human disease • Joint involvement unilateral
NHP (Rhesus macaques, Cynomolgus macaques)	• Evolutionary proximity—Similar physiology and immune response • Natural host • Replicates viremia, spread to organs and joints, fever, viral persistence, rashes, changes in blood biochemistry and CBC, cytokine, and chemokine response • Replicates joint involvement at very high challenge doses • Non-lethal	• Large animal model (size, generation time, handling, cost, etc.) • Ethical constraints of using primates in research • Replicates joint pathologies only at non-physiological challenge doses

The immune response to CHIKV infection involves both innate and adaptive immunity. Most infections begin with a mosquito bite delivering CHIKV to permissive cells in the skin, including epithelial cells, fibroblasts and macrophages (113). Virus spreads rapidly from the inoculation site to the circulatory system and further to multiple organs, with studies indicating that infiltration of organs is driven by infected monocytes/macrophages (114). The host immune system senses CHIKV presence, with multiple pattern recognition receptors playing a role, and mounts an antiviral response strongly dependent on type I interferons. The importance of these antiviral mediators is highlighted by animal experiments using mice deficient for components of type I interferon signaling, which are highly susceptible to CHIKV infection (107, 115–117). In addition, a variety of other cytokines and chemokines are induced upon infection, both in animal models and in humans undergoing natural infection, including IL-6, IL-1RA, IL-12, and CCL2 (118).

The immune response against CHIKV on a cellular level is only partially understood. Monocytes and macrophages appear to play an important role both in the control of infection as well as in associated pathology. Genetically depleting monocytes from mice leads to increased viremia in the footpad swelling model, indicating these cells are protective during infection (119). On the other hand, monocytes and macrophages are implicated in CHIKV associated joint pathologies. In Cynomolgus macaques, synovial macrophages were found to serve as a reservoir for CHIKV (111). In line with this observation, synovial isolates from patients contain high numbers of macrophages and monocytes. Synovial macrophages isolated from CHIKV infected patients further display an activated morphology (120) and the supernatant of primary human fibroblast-like synoviocytes challenged with CHIKV was shown to induce monocyte migration and drive the differentiation of monocytes/macrophages to osteoclast-like cells producing IL-6 and TNFα, both known for their involvement in arthritis (121). Interestingly, macrophage depletion in mice by means of chlondronate liposomes results in increased viremia, but decreased swelling in the footpad injection model, again highlighting the bivalent role of macrophages in CHIKV infection (122).

There is significant consensus within the scientific community that the induction of antibodies in general and neutralizing antibodies in particular comprises an immunological marker that likely correlates with protection against CHIKV infection and disease (2, 123, 124). Recent epidemiological studies conducted in the Philippines (125) and Cambodia (126, 127) have confirmed that (1) a positive baseline CHIKV plaque reduction neutralization titer is associated with 100% (95% CI 46.1–100.0) protection from symptomatic infection; (2) that broad cross-neutralization among CHIKV lineages, i.e., ECSA, WAf, Asian, IOL, exists; and that (3) it is highly likely that the elicitation of a neutralizing antibody response will provide very long-lasting (if not lifelong) immunity across all CHIKV genotypes. Similarly, in a study of serum antibodies from a 2008 outbreak in Singapore, the early induction of neutralizing antibodies correlated with rapid clearance of virus from the periphery and clinical protection against arthralgia. Findings regarding the importance of early neutralizing antibody responses in protection against arthralgia have also been recently documented in a prospective cohort in India (128). These observations are well-replicated in animal models. Passive transfer of IgG antibodies isolated from plasma of convalescent patients can efficiently prevent and cure CHIKV infection in mice (129). Additionally, treatment with neutralizing monoclonal antibodies (mAbs) specific for CHIKV E1 and E2 proteins protected IFNAR1^−/−^ mice against mortality and prevented development of chronic infection of Rag1^−/−^ mice lacking B and T cells. Similarly, passive transfer of monoclonal antibodies against CHIKV also protected NHPs from CHIKV challenge (130, 131). Taken together, data from humans, mice and NHP all suggest that neutralizing antibodies likely represent an immune correlate of protection.

The role of CHIKV-specific T cells is less clear. While CD4^+^ T cell help is undoubtedly necessary for the generation of protective antibody responses, these cells are also implicated in joint pathologies, probably best showcased by reduced footpad swelling in CD4^−/−^ mice. Interestingly, CD8 deficiency has limited impact on disease progression in the same study. Viremia is not affected by lack of T cells in mice (132). In contrast, a recent study found that a T-cell biased prophylactic vaccine approach was effective against CHIKV challenge in mice, indicating that T cell responses can contribute to protection (133).

### Past and Current Efforts in Vaccine Development

#### Zika Virus

The outbreak in Latin America in 2015 has driven the development of multiple vaccine candidates. Some of them have successfully completed the preclinical stage and have entered clinical trials. The most advanced vaccines currently in development are discussed below. Table 3 summarizes the candidates already in clinical development.

**Table 3:** ZIKV vaccine candidates in clinical development.

**Vaccine strategy**	**Candidate name**	**Sponsor**	**Non-clinical development**	**Phase 1**	**Phase 2**	**References**
DNA	GLS-5700	GeneOne Life Science, Inc. Inovio Pharmaceuticals vio	Immunogenicity in mice and NHP	NCT02809443 NCT02887482		(134, 135)
	VRC5283	NIAID/VRC	Immunogenicity in mice and NHP; efficacy in NHP	NCT02996461	NCT03110770	(136, 137)
	VRC5288			NCT02840487		
mRNA	mRNA-1325	Moderna Therapeutics	Immunogenicity and efficacy in mice	NCT03014089		(138)
	mRNA-1893		Efficacy in mice	NCT04064905		(139)
Whole inactivated	ZPIV	NIAID/WRAIR/BIDMC	Immunogenicity and efficacy in mice and NHP	NCT02963909 NCT02952833 NCT02937233 NCT03008122		(97, 98, 140)
	BBV121	Bharat Biotech International	Immunogenicity and efficacy in mice	CTRI/2017/05/008539		(141)
	PIZV (TAK-426)	Takeda Pharmaceuticals	Immunogenicity and efficacy in mice	NCT03343626		(142)
	VLA1601	Valneva Austria GmbH /Emergent Biosolutions		NCT03425149		
Live attenuated	rZIKV/D4Δ30-713	NIAID		NCT03611946		
Viral vectored	MV-ZIKA	Themis Bioscience GmbH	Immunogenicity in mice; efficacy in pregnant mice	NCT02996890		(143)
	MV-ZIKA RSP			NCT04033068		

##### DNA vaccines

Among the first candidates that have entered clinical trials in humans were DNA vaccines. Early after the onset of the Brazilian outbreak in 2015, research groups were focussed on the development of different DNA-based strategies including truncated prM protein, soluble E proteins and whole prM-E proteins and have compared the immunogenicity profile of the different constructs in mice (98). The animal studies have demonstrated the superiority of the constructs expressing the full-length prM and E sequence. The vaccine candidates that advanced to clinical trials are all based on the expression of prM-E in transfected cells, which spontaneously assemble into non-infectious sub-viral particles retaining structural similarity to native virions. The first vaccine candidate tested in clinical trials was GLS-5700 developed by Inovio Pharmaceuticals (NCT02809443 and NCT02887482). This DNA vaccine is based on a consensus prM-E sequence derived from African and more recent Asian/American strains downstream of the signal sequence of IgE. Preclinical studies in mice and NHP have confirmed its strong immunogenicity by showing prevention of viremia (134). Passive transfer of vaccine-induced antibodies into interferon α/β receptor–deficient mice protected mice from lethal challenge. Interestingly, when tested in humans, only 62% of the study participants developed neutralizing antibodies against ZIKV after receiving three doses of the vaccine candidate. The most frequent adverse events (AE) were mostly mild local injection site reactions, as well as headache and myalgia (135).

The Vaccine Research Center (VRC) and National Institute of Allergy and Infectious Diseases (NIAID) have developed two other DNA based vaccine candidates, VRC5283 and VRC5288. Both candidates encode a codon-optimized form of the prM-E sequence derived from the French Polynesian strain 2013 (136). The ZIKV prM signal sequence was replaced by a signal sequence from the Japanese encephalitis virus (JEV) prM protein. The usage of the JEV signal sequence should increase the signal peptide cleavage as previously demonstrated by studies with West Nile Virus (144). In VRC5288, the carboxyterminal stem-anchor region of ZIKV protein E was also exchanged to the equivalent JEV sequence in order to improve subviral particle release from transduced cells. Immunization of mice and NHPs demonstrated that both candidates VRC5283 and VRC5288 were able to elicit neutralizing antibodies after two administrations. Moreover, a ZIKV challenge study of previously immunized NHPs resulted in almost complete protection from viremia (136). Thus, various clinical Phase 1 trials with both candidates were initiated in the United States to evaluate different doses, dose regimens as well as delivery devices (NCT02996461 and NCT02840487). The most frequent AE were found to be mild to moderate local injection site reactions, as well as malaise and headache. VRC5283 revealed to be more immunogenic in humans with higher neutralizing antibody titers 26 days after the second vaccine administration compared to VRC5288 (137) and was thus moved forward to Phase 2 clinical studies (NCT03110770).

##### mRNA vaccines

Another appealing platform technology for the development of vaccines against infectious diseases is represented by mRNA vaccines. This technology has been improved over the last years by developing techniques to remove double stranded RNA product, by inserting modifications to increase RNA stability and by developing different formulations for delivery (145). In contrast to DNA which needs to enter the nucleus to start transcription, RNA can be directly translated in the cytoplasm upon cell transfection.

Diverse ZIKV mRNA vaccine candidates have been developed and studied in animals. One of the first was described by Pardi et al. (146). The mRNA candidate encodes the prM-E sequence of a French Polynesian strain. Wild type mice and NHP were protected from viremia after the administration of a single dose. Other similar mRNA vaccine candidates were developed by Moderna Therapeutics and are based on a prM-E sequence derived from the Micronesia 2007 strain. The engineered vaccine candidates only differ in their prM signal sequence expressing either the signal sequence of JEV or IgE. Testing in various mouse models revealed that both strategies resulted in immunogenic and efficacious vaccine candidates, albeit with some differences (138). mRNA-1325, a mRNA vaccine expressing the IgE signal sequence instead of the prM signal sequence, was selected as first candidate for further clinical development (NCT03014089). In parallel, preclinical development was continued and a second vaccine candidate mRNA-1893 was advanced to clinical studies entering in 2019 (NCT04064905). This vaccine candidate protected against ZIKV transmission during pregnancy in mice (139).

##### Whole inactivated vaccines

Whole inactivated vaccines have been successfully developed for other flaviviruses including TBEV and JEV. This approach was taken up by several groups. Immediately after the 2015 outbreak, the first preclinical studies using a purified inactivated ZIKV vaccine were described by Larocca et al. (98). The alum adjuvanted formalin-inactivated whole virus vaccine PRVABC59 which was derived from a strain from Puerto Rico protected mice from ZIKV challenge after a single immunization. A few months later, the same group confirmed the efficacy of this vaccine candidate also in rhesus macaques following two administration of the vaccine candidate 1 month apart. Protection was confirmed using different ZIKV challenge strain—Brazilian and Puerto Rico ZIKV isolates (97). In addition, two doses of PRVABC59 protected rhesus monkeys even when challenged 1 year later. The Walter Reed Army Institute of Research developed this vaccine candidate further, under the name ZPIV. The safety and immunogenicity of the ZPIV was tested and confirmed in three placebo-controlled trials (NCT02963909, NCT02952833, and NCT02937233). Besides mild to moderate injection site reactions the most frequent systemic AEs observed were fatigue, headache, and malaise. Passive transfer of purified IgG from immunized recipients into immunocompetent mice reduced the viral loads upon ZIKV challenge (140). A fourth trial in a Flavivirus endemic area is still ongoing (NCT03008122). In 2016, a research agreement was signed between WRAIR and Sanofi Pasteur, with the latter taking over all further non-clinical and clinical development efforts. A modified Zika vaccine candidate (ZIPV-SP) was developed by Sanofi Pasteur which demonstrated higher immunogenicity and efficacy in mice compared to the first-generation vaccine ZPIV (147). This candidate will advance to further clinical trial testing in future.

Bharat Biotech International (India) has started its ZIKV vaccine development after the French Polynesian outbreak in 2013. In preclinical mice studies using immunodeficient AG129 mice, the formalin-inactivated whole virus vaccine (BBV121) demonstrated its immunogenic potential by protecting against Asian and African challenge strains (141). BBV121 is currently assessed in a Phase 1 clinical trial in India (CTRI/2017/05/008539). Similar approaches using formalin inactivated whole viruses as vaccine candidates are currently pursued by Takeda Pharmaceuticals and Valneva Austria GmbH/Emergent BioSolutions (NCT03343626 and NCT03425149, respectively). PVIZ (TAK-426) developed by Takeda conferred protection against lethal challenge in mice (142). No pre-clinical data have been yet reported for the Valneva/Emergent BioSolutions vaccine candidate.

##### Live attenuated vaccines

Vaccination experiences with other live attenuated flaviviral vaccines like YFV and JEV propose that a live attenuated ZIKV vaccine could be a promising approach for generating a robust immune response. In contrast to the traditional approach used for the 17D YF vaccine in which attenuation was achieved by several passages on different animal tissues, ZIKV attenuation was achieved by direct manipulation of the viral genome. This was only possible due to the fact that ZIKV infectious cDNA clones were available shortly after the first outbreaks (148). Attenuation approaches include the removal of NS1 carbohydrates, site-directed mutagenesis of the 3′-UTR or the formation of chimeric flaviviruses encoding the ZIKV prM and E sequence in the context of an attenuated heterologous background.

Attenuated viruses carrying a 10 nucleotide deletion in the 3'UTR of a Cambodian Strain provided sterilizing immunity in A129 mice and rhesus macaques (149). Further analysis with this vaccine candidate in pregnant C56BL/6 mice in which the IFNAR1 receptors were blocked by antibody treatment revealed that a single dose is able to significantly reduce vertical transmission and prevented damage of the testis. A similar vaccine construct carrying a larger deletion in the 3′UTR was also shown to be efficacious in animal studies, already at low doses (150).

Xie et al. at the University of Texas Medical Branch have been involved in the generation of a chimeric virus containing the Zika prM-E in a Dengue Virus 2 (DENV-2) backbone. The chimeric vaccine protected A129 mice against ZIKV challenge (151). A second chimeric vaccine candidate using the ZIKV prME proteins in a DENV-4 backbone encoding also a 30 nucleotide deletion in the 3′ UTR region has been developed by NIAID. This vaccine candidate was tested recently in a Phase 1 clinical trial (NCT03611946).

##### Viral vectors

Another vaccine approach evolved over the last decades deals with the expression of ZIKV genes in the context of viral vectors, either replication competent or defective. Different viral vectors including adenovirus, vesicular stomatitis virus (VSV), vaccinia virus, or measles vaccine virus are used as a delivery platform for the production of heterologous antigens upon cell infection, making them a powerful plug and play technology for the rapid development against emerging diseases.

A rhesus adenovirus based vaccine candidate (RhAd52) expressing the ZIKV prM-E proteins was assessed for its immunogenicity and efficacy in rhesus monkeys (97). This vaccine candidate elicited high neutralizing antibody titers and prevented ZIKV from viral replication upon challenge. Interestingly, a single immunization induced a robust protection against ZIKV challenge in rhesus macaques 1 year after the administration (152). Several other adenovirus-based vaccine vectors using other backbones are in pre-clinical development and have proven their immunogenic capacity in mice (153–157).

A live attenuated measles Schwarz vaccine vector expressing the ZIKV prM-E was developed by Themis Bioscience and was evaluated in a Phase 1 clinical trial in Austria (NCT02996890). Preclinical studies in an allogenic mouse pregnancy model have shown that vaccination with this candidate reduced the ZIKV load in distinct organs and prevented fetal infection (143). Recently, a second measles-based ZIKV vaccine candidate developed by Themis-Bioscience entered Phase 1 clinical trials (NCT04033068).

Besides adeno- and measles virus-based vectors, a further promising vaccine candidate based on a vaccinia virus vector directed against both emerging diseases ZIKV and CHIKV was successfully tested in preclinical studies. The vector has incorporated ZIKV prM-E as well as the structural proteins of CHIKV. A single immunization of this multivalent vaccine induced neutralizing antibodies toward both viruses in mice and protected against CHIKV viremia and arthritis as well as ZIKV viremia and fetal/placental infection or testis infection (156).

Another approach utilizes recombinant VSV vectors expressing ZIKV prM-E proteins. One candidate showed a good humoral and cellular T cell response in wild-type mice, another protected from lethal challenge (158, 159).

##### Virus-like particle vaccines

*In vitro* purified virus-like particles (VLP) represent an alternative approach for the development of a ZIKV vaccine. As already mentioned above expression of ZIKV structural proteins give rise to the development of non-infectious virus-like particles which present antigens in their native confirmation leading to the development of high neutralizing antibody titers. VLP vaccines are efficiently produced by generation of stable cell lines. Some groups have explored their immunogenic potential in preclinical animal studies (160, 161). In an alternative approach, a recent study used bacteriophage VLP platforms displaying predicted Zika B cell epitopes and showed immunogenicity in mice (85).

A wide variety of promising vaccine candidates for the prevention of Zika induced disease have undergone pre-clinical and early clinical evaluation, many of which would certainly be suitable for further clinical development. The impressive speed with which these candidates were generated, tested in animals and in several cases brought to first-in-man trials highlights how quickly manufacturers and developers can react to large scale outbreaks. In the wake of the large Zika epidemic public funding for vaccine development has largely expired and development efforts have slowed. Together with regulatory challenges associated with the development of vaccines against diseases with unpredictable epidemiology, this represents a major hurdle in bringing promising candidates to licensure.

#### Chikungunya Virus

The circulating genotypes of CHIKV are genetically closely related and appear to constitute a single serotype. Moreover, infection with CHIKV causes long-lasting, possibly life-long protection (126, 128, 162, 163). Taken together, these observations suggest the development of a vaccine as a promising route for the prevention of Chikungunya fever. Several groups have initiated the development of prophylactic vaccine candidates against CHIKV and have started evaluation in preclinical studies as well as Phase 1 and Phase 2 clinical trials. A summary of the most advanced vaccine candidates in clinical development is given in Table 4.

**Table 4:** CHIKV vaccine candidates in clinical development.

**Vaccine strategy**	**Candidate name**	**Sponsor**	**Non-clinical development**	**Phase 1**	**Phase 2**	**References**
Whole inactivated	–	USAMRIID and WRAIR	Immunogenicity and efficacy in mice and NHP	Completed		(164)
	BBV87	Bharat Biotech International	Immunogenicity and efficacy in mice	CTRI/2017/02/007755		(165)
Live attenuated	TSI-GSD-218 (181/clone 25)	Unites States Army Medical Research Institute of Infectious Diseases	Immunogenicity and efficacy in mice and NHP		Completed	(166–168)
	VLA1553	Valneva Austria GmbH	Immunogenicity and efficacy in mice and NHP	NCT03382964		(169–171)
Viral vectored	MV-CHIK	Themis Bioscience GmBH	Immunogenicity and efficacy in NHP	EudraCT-2013-001084-23	NCT02861586 NCT03101111 NCT03028441 NCT03635086 NCT03807843	(109, 172–174)
	ChAdOx1 Chik	Jenner Institute, University of Oxford	Immunogenicity and efficacy in mice	NCT03590392		(175, 176)
Virus like particles	VRC-CHKVLP059-00-VP	NIAID	Immunogenicity and efficacy in mice and NHP	NCT01489358 NCT03028441	NCT02562482	(108, 177, 178)
	PXVX0317 (former name: VRC-CHKVLP059-00-VP)	Emergent BioSolutions			NCT03483961	
mRNA	VAL-181388	Moderna Therapeutics	Immunogenicity and efficacy in mice; immunogenicity in NHP	NCT03325075		

##### Whole inactivated vaccines

The US Army Medical Research Institute of Infectious Diseases (USAMRIID) together with WRAIR developed a formalin-inactivated CHIKV vaccine which showed encouraging immunogenicity and efficacy data in mice and NHP using four different CHIKV strains (179). In further instance, the candidate was tested in 16 healthy adults and found to be immunogenic and well tolerated in human, with no AEs reported in any of the participants (164). Despite these promising results, the program was discontinued. Two recent candidates based on chemical inactivation of whole virus were assessed in mouse models successfully demonstrating their immunogenic potential (180), with one of these vaccines also able to protect vaccinated animals from CHIKV challenge (165). This latter candidate was recently advanced to clinical phase 1 evaluation in India by Bharat Biotech International (CTRI/2017/02/007755).

##### Live attenuated vaccines

Development of a live-attenuated vaccine initiated by USAMRIID yielded the strain CHIK 181/clone 25 (181). This candidate was protective in mice and revealed reduced virulence in monkeys. Furthermore, 181/25 underwent clinical evaluation and showed promising immunogenicity and a largely acceptable safety profile in human, with largely mild injection site reactions reported in 20% of participants, and the most frequent systemic AE represented by flu-like symptoms, transient arthralgia and urticaria. Transient arthralgia was the only AE found more frequently in actually vaccinated participants, as compared to the placebo group, which raised some concerns (166). A later study showed that attenuation of this strain is based on only two point-mutations suggesting a tangible risk for reversion to virulence (182). Thus, the development of the candidate was discontinued in 1998 and the strain was later made available to developers (167).

Targeted attenuation of CHIKV by means of genetic engineering gave rise to several other promising candidates. Partial deletion of the gene encoding the non-structural protein nsP3 resulted in a live-attenuated vaccine showing immunogenicity and efficacy in the mouse footpad swelling model (169, 170) and in Rhesus macaques (171), and has been evaluated in a recently concluded phase 1 clinical trial by Valneva Austria GmbH (NCT03382964). While results have not yet been published, safety and immunogenicity data were presented at recent conferences and look promising.

An additional attenuated CHIKV strain derived from La Reunion was generated by University of Texas Medical Branch through replacing the subgenomic promoter for expression of the structural genes with an internal ribosomal entry site derived from ECMV. This construct was highly immunogenic and protective in the IFNAR1^−/−^ mice and Cynomolgus macaques (183–185).

##### Viral vectors

The excellent efficacy and safety of viral vectored vaccines is well-established. The use of viral vectors is a potent tool in gene therapy and vaccines development due to the ability to induce both potent humoral and cellular immune responses. The immunogenicity is further enhanced through intrinsic vector motifs that stimulate the innate immunity pathways (186, 187). Thus, the use of expensive and mostly reactive adjuvants can be omitted. Viral vectors can use the host-cell protein-processing pathways that lead to antigen presentation via the MHC I complex and consequent cytotoxic T-cell stimulation (188). In addition, viral vectors can be produced in high quantities at relatively low costs, which allows the use of these systems in low-income countries.

The currently most advanced vaccine in development is the live-recombinant measles vectored vaccine MV-CHIK that is based on the Measles Schwarz vaccine strain. The vector is genetically modified to express the full subgenomic RNA encoding the structural proteins, while the measles backbone remains unchanged and functional. Studies in a measles virus susceptible mouse model—hCD46/IFNAR^−/−^ mice—showed the immunogenicity and efficacy of the vaccine after one or two doses (109). Furthermore, the efficacy was demonstrated in cynomolgous macaques. All animals that received two full human vaccine doses elicited high levels of neutralizing antibodies that were cross-reactive to all circulating CHIKV lineages. The animals were fully protected against CHIKV disease manifestations including viremia and fever (174). The MV-CHIK vaccine was safe and well-tolerated in phase 1 and phase 2 clinical studies (EudraCT 2013-001084-23 and NCT02861586, respectively) and found to be highly immunogenic as determined by the induction of functional, neutralizing antibodies. The most frequently observed AEs were mostly mild to moderate injection site reactions as well as fatigue and headache. Interestingly, the measles vectored vaccine was effective even in the presence of pre-existing anti-vector immune responses (172, 173). Several other clinical trials are currently ongoing (see Table 2).

MVA-based CHIKV vaccine constructs expressing CHIKV inserts derived from the S27 strain were assessed for efficacy in small animal models. Different compositions of the antigenic structures (6KE1, E3E12, or the entire envelope protein cassette E3-E2-6K-E1) induced neutralizing antibodies to the homologous CHIKV S27 strain in immunocompromised AG129 mice. However, titers elicited by the MVA-6KE1 and MVA-E3E2 were significantly lower than titers induced by the full envelope cassette. Interestingly, both the MVA-E3E26KE1 and MVA-E3E2 fully protected the CHIKV susceptible AG129 mice against lethal challenge with CHIKV S27 strain (189). Similar findings were made in another lab with a recombinant MVA construct expressing E3-E2 only. The co-expression of E3 peptide together with E2 facilitates correct folding of the E2 protein. Wild type BALB/c mice immunized with the MVA-E3-E2 were protected from viremia. Additionally, A129 mice lacking the Type 1 IFN receptor were protected from viremia, footpad swelling, and mortality (190). Furthermore, a MVA-vectored vaccine candidate with an E3-E2-6K-E1 CHIKV insert was derived from the Indian Ocean strain LR2006-OPY. This vaccine strain induced very high titers of neutralizing antibodies and a strong cellular CD8^+^ T-cell response. The MVA-CHIK vaccine protected C57BL/6 mice against challenge in a footpad-swelling model (191). All MVA-based CHIKV candidates described here are immunogenic and protect against CHIKV challenge in different mouse models. However, none of the candidates progressed to clinical development.

Another candidate was created by replacing the glycoprotein G of Vesicular Stomatitis Virus (VSV) with the CHIKV structural cassette, resulting in a chimeric virus shown to be immunogenic and protective in the mouse footpad swelling model. In addition, the VSV-CHIK constructs induced a CHIKV E1- and E2-specific IFNγ-producing T-cell response after a single immunization (192).

Two further pre-clinical candidates were created based on adenoviruses. One study showed that a vaccine based on the non-replicating Complex Adenovirus vaccine and expressing the CHIKV structural polyprotein induced antibodies and protected mice in the footpad swelling model (193). Another construct was generated based on the Chimpanzee adenoviral vector platform ChAdOx1. A mosaic consensus CHIKV structural gene sequence was inserted in the ChAdOx1. The mosaic consensus sequence was derived from the Asian, ECSA and West African lineages that potentially provides a broad protective range over all genetic lineages of CHIKV. The recombinant vaccine indicated a strong cellular and humoral response in BALB/c mice. Interestingly, the 6K protein represented the immunodominant peptide to induce T cell responses, the humoral response is directed mainly to the E2 antigen. The use of a heterologous prime boost vaccination scheme composed of a recombinant ChAdOx and MVA enhanced the vaccine immune response in the BALB/c animal model (176). In addition, the ChAdOx vaccine protected from lethal challenge in the immunodeficient A129 mouse model (175). The Jenner Institute, University of Oxford has assessed the immunogenicity and safety of a single dose ChAdOx1 CHIK vaccine in a phase 1 clinical trial which has recently been completed (NCT03590392).

Using an attenuated strain of Venezuelan equine encephalitis virus (VEEV) or Sindbis virus as backbone to generate a potent CHIKV vaccine has been used by the University of Texas Medical Branch. The structural proteins of VEEV or Sindbis were replaced by the structural proteins of CHIKV, rendering the virus highly attenuated. Immunization of mice resulted in protection from viremia post-challenge (194). One of the candidates, VEEV-CHIKV was later modified by replacing the subgenomic promotor with an IRES element (195), thereby increasing its safety profile.

Lastly, a recent study showed immunogenicity and protective capacity of a CHIKV vaccine based on the Eilat virus, an alphavirus host-restricted to insects. In this construct, the structural genes of the parental Eilat virus are replaced by the CHIKV structural genes, resulting in a chimeric virus unable to replicate in vertebrate cells. This vaccine candidate was assessed in IFNAR1^−/−^ mice as well as in the mouse footpad swelling model and Cynomolgus macaques and found to be protective (196).

##### Virus-like particle vaccines

CHIKV virus like particles (VLP) represent an optimal antigenic structure without exposing the vaccine to replicating CHIKV. The epitopes are presented in the correct structure for immune recognition. A VLP vaccine program was developed at the vaccine research center (VRC) at NIH. The vaccine candidate VRC-CHKVLP059-00-VP is comprised of VLPs expressed from human embryonic kidney cells transfected with plasmid encoding all structural protein genes (C-E3-E2-6K-E1) from CHIKV strain 37997 (West African lineage). In cynomolgus monkeys, these VLPs were highly immunogenic and the antibodies produced were cross reactive to all CHIKV lineages (108). This candidate was moved toward phase 1 clinical trial (NCT01489358 and NCT03028441), which confirmed the vaccine immunogenicity and safety (40) as well as the induction of broadly cross-neutralizing antibodies (178). Only mild local injection site reactions were reported, the most frequent systemic AEs observed were malaise, headache and nausea. As a next step, the vaccine was further assessed in Phase 2 clinical trials—one completed (NCT02562482) and one still ongoing (NCT03483961)—by NIAID and Emergent Biosolutions. The data have not been published yet but presented at various conferences (including ASTMH 2019). The vaccine induced potent immune responses with and without the presence of the vaccine adjuvant alum.

Other approaches for the production of enveloped VLPs include the use of insect cells or yeast instead of mammalian cells in order to increase the yield which is a limiting factor in manufacturing. VLPs expressed by a baculovirus platform were immunogenic in mice and provided full protection against viremia and inflammation in joints upon challenge (197). Yeast-derived VLPs showed efficient *in vitro* and *in vivo* neutralization activity and conferred protection in CHIKV infected neonatal mice (198). Bacteriophage VLPs displaying immunogenic peptides represent an alternative approach; a recent publication showed that E2 derived B cell epitopes displayed in such a manner can induce neutralizing antibodies (199).

##### DNA vaccines

DNA vaccines are easy and cost-effective to manufacture and require less stringent cold-chain storage conditions. However, the use of a medical device required for vaccine administration is required. Several DNA vaccine strategies were developed in the last decade. The first developed CHIKV DNA vaccine candidate pMCE321 encode the structural genes C, E2, and E1. The genetic information was derived from a consensus sequence of multiple NCBI strains. In preclinical mouse and monkey studies, the vaccine elicited neutralizing antibodies as well as CD8^+^ T cell responses (200). Modification of the DNA vaccine candidate by addition of a nsP2 sequence improved its immunogenicity (201). The next generation of CHIKV DNA vaccines used the whole CHIKV genome of the 2006 OPY1 strain carrying either a deletion of the 6K gene or parts of the nsP3 protein. These vaccines were also highly immunogenic and protected wild-type C56BL/6 mice from viremia and footpad swelling (169). A DNA vaccine (DRP-E) carrying a deletion of the gene coding for the capsid protein C represents another DNA based approach, also referred to as a DNA-launched replicon vaccine. Replicons are self-replicating RNAs that cannot assemble to infectious virus particles due to the lack of structural proteins. As DRP-E only expresses the CHIKV envelope proteins (E3, E2, 6K, and E1), no nucleocapsid and consequently no virions can be formed within transfected cells. This CHIK replicon vaccine was also found to be immunogenic and protective in mice and NHP (170, 171).

An additional strategy is the generation of infectious virions of the live-attenuated 181/25 strain from a DNA plasmid (iDNA) by usage of a cytomegalovirus promotor. In BALB/c mice, this iDNA vaccine induced neutralizing antibodies and protected from viremia upon challenge (202). Interestingly, the reversion rate of attenuating mutations was much lower as compared to the 181/25 parental vaccine candidate, further suggesting a better safety profile of DNA-launched infectious particle vaccines compared to the common live attenuated options.

##### mRNA vaccines

mRNA vaccines represent one of the newest strategies in the development of vaccine candidates against infectious diseases which are comprised of *in vitro* transcribed RNA. One mRNA based prophylactic vaccine candidate VLA-181388 was developed by Moderna Therapeutics. The company has publicly announced that a single dose induced protection against challenge in mice and neutralizing antibodies in NHP. So far, no pre-clinical data have been published. Currently, a Phase 1 clinical trial is evaluating the safety and immunogenicity of this candidate (NCT03325075).

##### Subunit vaccines

Subunit vaccines generally have a good safety profile compared to other vaccine candidates as no viral DNA/RNA or infectious particles are present. In addition, the production of single proteins is scalable and can be easily adapted to large-scale manufacturing purposes. A disadvantage of this vaccine candidate may be the limited immunogenic potential due to the fact that quaternary epitopes are not present. In addition, subunit vaccine necessitates the use of vaccine adjuvants that potentially increase production costs. Different groups have followed this strategy and have generated E1 and E2 subunit vaccines by the baculovirus system or by bacterial expression system (165, 203). Bacterially expressed E1 and E2 proteins elicited good humoral response and a balanced Th1/Th2 response in BALB/c mice, albeit strongly adjuvant-dependent (204). Recombinant E1 and E2 generated from insect cells induced neutralizing antibodies in AG129 mice. Nevertheless, when compared to VLPs, the subunit glycoproteins E1 and E2 were less immunogenic in a lethal mouse model (205).

Four vaccine candidates for the prevention of Chikungunya disease have progressed past phase 1 clinical studies, all of which look promising in terms of safety and immunogenicity. While the candidates are based on very different platform technologies, they all induce neutralizing antibodies, a reasonably likely correlate of protection. Thus, all these candidates are probably suitable for the prevention of Chikungunya disease. However, the unpredictable epidemiology of CHIKV remains a challenge in bringing any of these candidates to licensure, as discussed in greater detail below.

## Regulatory Progress and Health Policy Framework in The Vaccine Development for Zika and Chikungunya Virus

The development of safe and effective vaccines suitable for the use in all age groups is of utmost importance to prevent the spread of CHIKV and ZIKV and for successful outbreak intervention. Regulatory bodies including European Medicines Agency (EMA) and the US Food and Drug Administration (FDA) agree on the urgent medical need for CHIKV and ZIKV vaccines (123, 206). The EMA has granted PRIority MEdicine status (PRIME) to two Chikungunya and one Zika vaccine candidate in development. The US FDA has designated three CHIKV vaccine and three ZIKV vaccine candidates with a Fast Track status. These programs consider the urgent medical demand and the advanced development status of these specific vaccine candidates and will facilitate and accelerate the development and licensure of these vaccines. In addition, large international funding organizations including the US Biomedical Advanced Research and Development Authority (BARDA), the EU Commission framework Horizon 2020 and many national funding agencies have dedicated substantial funding opportunities for developers. Another major milestone for the development of emerging infectious disease vaccines including CHIKV vaccines was the formation of the Coalition for epidemic preparedness (CEPI) in the aftermath of the 2014-15 Ebola outbreak. Major funding and development efforts at CEPI were dedicated to the licensure of CHIKV vaccines (207). FDA has previously implemented an incentive program that aims to facilitate the development of drugs and vaccines against diseases not profitable for developers, including neglected tropical diseases. The “priority review voucher” is awarded to the sponsor of a newly approved drug and entitles to get priority review for another product. In 2018, Chikungunya was added to the list of eligible diseases, highlighting the recognition of an urgent unmet medical need for a Chikungunya vaccine.

The development of vaccines from early development to the availability on the market is a timely process that can take more than 10 years with cost >$100 Million US Dollars (208). The licensure process is defined by EMA and FDA based on requirements that are either provided in the code of federal regulation 21 (CFR 21) or in guideline documents on the clinical evaluation of vaccines (EMEA/CHMP/VWP/164653/05). In traditional licensure procedures, the vaccine safety has to be recorded in a sufficiently sized safety database. The vaccine efficacy is typically demonstrated in randomized, controlled vaccine efficacy phase 3 trials in affected populations in areas with sufficient disease transmission (209). However, outbreak viruses like CHIKV and ZIKV show a highly unpredictable epidemiology. Both viruses caused a few major (>100.000 cases) and many small outbreaks (>100 cases) in the last two decades (15). The outbreaks are typically short in duration and the case numbers wane within very few weeks or months. In addition, many affected areas in the tropical and sub-tropical regions of the world might not have a sophisticated disease reporting, diagnostic or surveillance system. In most of these countries other febrile illnesses co-circulate including Dengue or Malaria, which can often lead to misdiagnosis. In addition, the report of cases is often based on clinical diagnosis with the lack of serological confirmation. Taken together, these factors lead to unreliable assessment of disease incidence. The study design of a randomized controlled efficacy clinical trial is based on disease surveillance data. To allow for a statistically meaningful outcome, the sample size of the clinical trial is determined by the incidence of the disease in the study population and more specifically, the number of detected cases. Thus, the planning and conduct of randomized controlled clinical trial to show ZIKV and CHIKV vaccine efficacy is not feasible. In the FDA CFR21 and the EMA guideline for evaluation of vaccines it is foreseen that alternatives are acceptable if a pre-licensure clinical efficacy study is not feasible. Technically, the licensure pathways are different in the respective regions. However, the fundamental requirements are similar. The licensure can be based on an immunological marker that is reasonably likely to correlate with protection (surrogate marker). In addition, the use of well-characterized animal models that are suitable to reflect human disease outcomes can support vaccine licensure (123, 206). The requirements on either strategy or a combination of both is individually assessed and is based on the antigen used and the method of vaccine delivery (i.e., vaccine technology).

The discussions between vaccine developers and regulatory bodies have substantially advanced in the last few years. The FDA recently engaged with the Vaccine & Related Biological Products Advisory Committee (VRBPAC) to discuss topics on the licensure of CHIKV vaccines. The purpose of the meeting was to publicly discuss the feasibility of vaccine efficacy trials and use of a non-human primate animal model to assess vaccine efficacy. The recently published meeting minutes state that the epidemiology of CHIKV does not allow for the conduct of randomized controlled clinical efficacy trials in the immediate future, and the combination of seroepidemiological knowledge can be combined with animal models to identify an immune marker reasonably likely to predict vaccine effectiveness are supported (https://www.fda.gov/advisory-committees/advisory-committee-calendar/vaccines-and-related-biological-products-advisory-committee-november-8-2019-meeting-announcement). In addition, the WHO has assembled experts in for a R&D blueprint meeting on the same topic. The experts gave guidance on current epidemiological features of the disease and on potential clinical trial designs (210).

In 2016, the WHO declared a public health emergency during the ZIKV outbreak in the Americas. To facilitate continued ZIKV vaccine development the WHO and NIH/NIAD co-hosted an expert and regulatory meeting in 2018. The experts concluded that vaccine efficacy trials are not feasible, and a discussion was held on the use of immunological markers that predict protection (206). Taken together, these public discussions are important to get a common understanding and opinion on the critical steps toward vaccine licensure (123, 206). Several CHIKV and ZIKV vaccines are in late stage clinical development. Ongoing clinical development strongly suggests that several candidate vaccines are suitable for the prevention of ZIKV and CHIKV disease, both in terms of safety and immunogenicity. Taken together, the public discussions described above have improved clarity on how such vaccines can get to licensure in the face of unpredictable epidemiology. Thus, there is cause for optimism that vaccines will be available in the near future, and that their path to licensure might serve as a blueprint for future vaccines to prevent emerging diseases.

## Author Contributions

SS mainly contributed on the Zika virus and Zika vaccine section by literature review, manuscript writing, and final editing. RT mainly contributed to the Chikungunya virus and Chikungunya vaccine sections by literature review, manuscript writing, and final editing. ET reviewed the content and contributed to Zika and Chikungunya vaccine development status. KR contributed to the regulatory review and introduction, and final review of the manuscript content.

### Conflict of Interest

All authors are employees of Themis Bioscience GmbH, a company developing vaccines to prevent Chikungunya and Zika.
